# Development of lentiviral vectors for efficient glutamatergic-selective gene expression in cultured hippocampal neurons

**DOI:** 10.1038/s41598-018-33509-5

**Published:** 2018-10-11

**Authors:** Yoshihiro Egashira, Yasunori Mori, Yuchio Yanagawa, Shigeo Takamori

**Affiliations:** 1 Laboratory of Neural Membrane Biology, Graduate School of Brain Science, Doshisha University, 1-3 Tatara Miyakodani, Kyotanabe, Kyoto 610-0394 Japan; 2 Department of Genetic and Behavioral Neuroscience, Graduate School of Medicine, Gunma University, 3-39-22 Showa-machi, Maebashi, Gunma 371-8514 Japan; 3 Present Address: Department of Physiology, Faculty of Medicine, Osaka Medical College, 2-7 Daigaku-machi, Takatsuki, Osaka 569-8686 Japan

## Abstract

Targeting gene expression to a particular subset of neurons helps study the cellular function of the nervous system. Although neuron-specific promoters, such as the synapsin I promoter and the α-CaMKII promoter, are known to exhibit selectivity for excitatory glutamatergic neurons *in vivo*, the cell type-specificity of these promoters has not been thoroughly tested in culture preparations. Here, by using hippocampal culture preparation from the *VGAT-Venus* transgenic mice, we examined the ability of five putative promoter sequences of glutamatergic-selective markers including synapsin I, α-CaMKII, the vesicular glutamate transporter 1 (VGLUT1), Dock10 and Prox1. Among these, a genomic fragment containing a 2.1 kb segment upstream of the translation start site (TSS) of the *VGLUT1* implemented in a lentiviral vector with the Tet-Off inducible system achieved the highest preferential gene expression in glutamatergic neurons. Analysis of various lengths of the VGLUT1 promoter regions identified a segment between −2.1 kb and −1.4 kb from the TSS as a responsible element for the glutamatergic selectivity. Consistently, expression of channelrhodopsin under this promoter sequence allowed for selective light-evoked activation of excitatory neurons. Thus, the lentiviral system carrying the VGLUT1 promoter fragment can be used to effectively target exogenous gene expression to excitatory glutamatergic neurons in cultures.

## Introduction

The mammalian central nervous system (CNS) consists of two major classes of neurons, glutamatergic excitatory neurons and GABAergic inhibitory neurons. In addition to the opposing effects on circuit activity, these two neuronal subtypes show many differences in physiological properties^1^. Thus, methods that enable genetic targeting of either cell type have been useful in numerous studies investigating the cellular functions as well as the roles of these cells in the neural networks.

Targeted gene manipulation in specific neuronal subpopulations has been achieved through either transgenic or viral approaches^2^. Although the transgenic approach is genetically specific, due to the acceptance of large genomic DNA or regulatory elements, it generally requires the generation and maintenance of a new animal line for each experimental aim. In contrast, viruses are readily adaptable to diverse genetically-encoded tools and thus now are being used widely in biological research^3^. Among the available viral vectors, self-inactivating lentiviral vectors are one of the most promising tools for gene delivery to neurons as they allow for stable, long-lasting transgene expression in post-mitotic non-dividing cells without significant toxicity^4^. However, due to the limited capacity of the vector (~8 kilobases (kb)), the internal promoter fragment that drives the gene expression must be short, thereby making it challenging to obtain strong and cell type-specific expression.

Attempts to establish an experimental tool for the genetic manipulation of neuronal cells *in vivo* revealed that lentiviral vectors equipped with either 0.4–1.1 kb fragments of the synapsin I promoter region or a ~1.3 kb fragment of the α-CaMKII promoter region effectively target gene expression to cortical neurons in the intact rodent brain^5–7^. Interestingly, it appears that both promoters show a strong preference for excitatory neurons, with weak to no expression in inhibitory interneurons^5–7^. However, one of these studies demonstrated that adeno-associated viruses (AAV), but not lentiviruses, carrying the synapsin I promoter efficiently produce reporter expression both in cortical excitatory and inhibitory neurons, highlighting the importance of the endogenous tropism of the viruses employed for cell type-specific gene expression^6^. An additional drawback of these cell type-specific promoters is their weak transcriptional activities compared to the transcriptional activities of other ubiquitous promoters. This weak transcriptional activity has hampered the widespread application of these promoters^8,9^. To overcome this limitation, several groups have exploited the Tet-Off inducible system in combination with established lentiviral vectors resulting in highly efficient transgene expression in a neuron-specific manner^10–12^.

Although the neuronal subtype-specificity of these viral promoters has been well characterized in the brain *in vivo*, it is not clear whether this specificity is preserved in primary neuronal cultures, since the gene expression pattern in developing cultured neurons may not precisely reflect that observed in the intact brain of adult animals. In the present study, we investigated the cell type-preference of the synapsin I and the α-CaMKII promoter in lentiviral vectors carrying the Tet-Off system in cultured hippocampal neurons. We found that these two promoters did not show adequate preference for glutamatergic neurons in the culture preparations; thus, we further explored putative promoter regions of three additional genes that are known markers of glutamatergic neurons in the hippocampus. We demonstrated that a 2.1 kb sequence upstream of the translation start site (TSS) of *VGLUT1*^13,14^ act as a promoter with a strong preference for glutamatergic neurons.

## Results

### The promoter for *VGLUT1*, but not *synapsin I* or *α-CaMKII*, showed preference for excitatory neurons in primary hippocampal cultures

**Figure 1:**
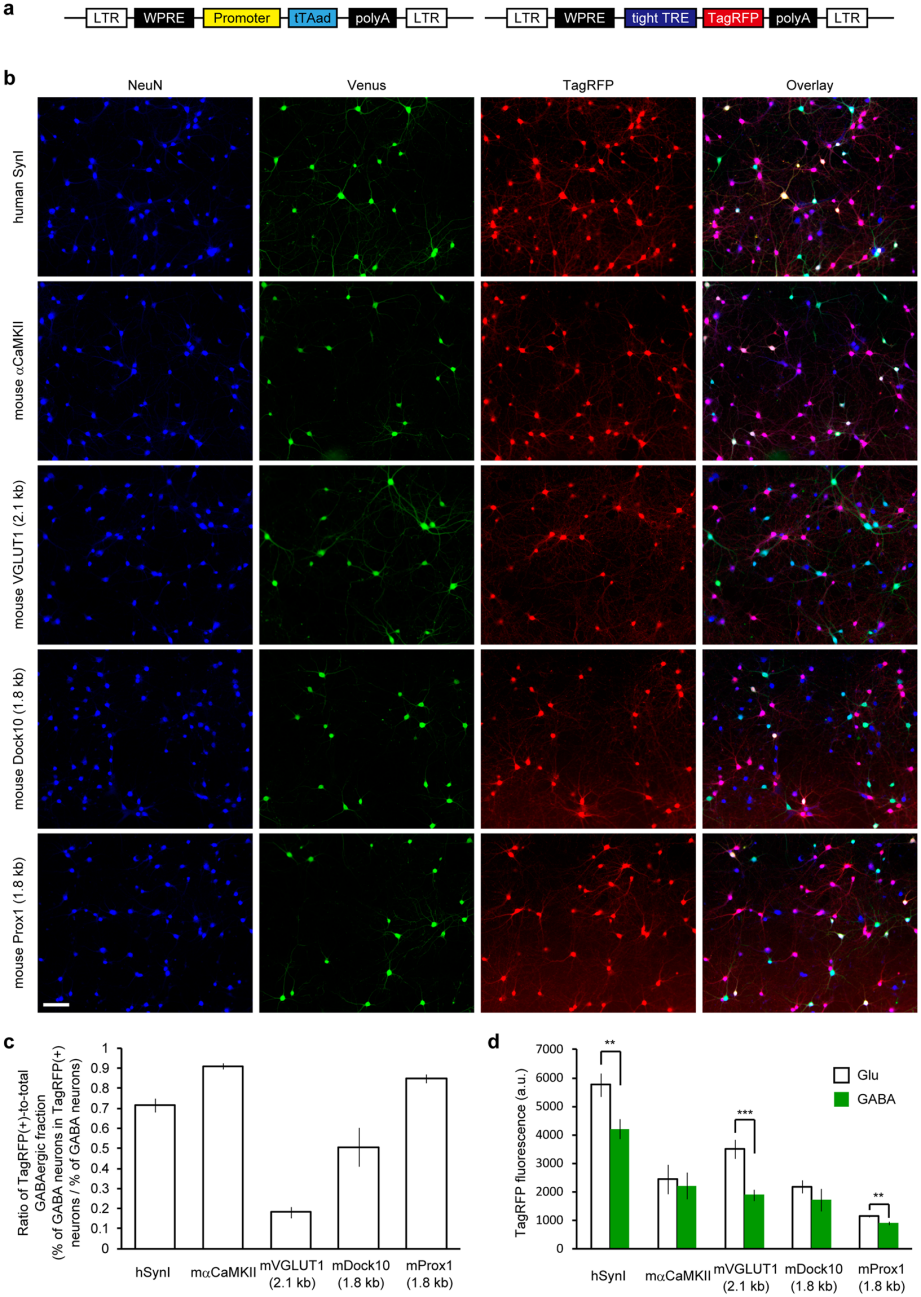
Glutamatergic neuron-specificity of the five different lentiviral promoters assessed in cultured hippocampal neurons from *VGAT-Venus* Tg mice. (**a**) Schematic drawing of a pair of the lentiviral vectors that depend on the Tet-Off system to drive TagRFP expression under the promoters tested in this work. Transgene sequences flanked by long terminal repeat (LTR) sequences, which facilitate the integration into the host genome, are shown. A regulator vector (left) expresses an advanced tetracycline transactivator (tTAad) under the control of a given promoter and a response vector (right) expresses TagRFP in the presence of tTAad. See the Materials and Methods section for details. (**b**) Fluorescence images of cultured *VGAT-Venus* Tg neurons virally-expressing TagRFP using the five different promoters. Neuronal somata are indicated by anti-NeuN immunostaining (blue). Venus fluorescence, amplified by anti-EGFP immunostaining, indicates GABAergic neurons (green). TagRFP fluorescence indicates reporter expression (red). Note that TagRFP-positive GABAergic neurons, indicated by a white appearance in the merged images are rarely observed in the VGLUT1 promoter condition. Scale bar indicates 100 μm. (**c**) The ratio of TagRFP-positive populations within GABAergic neurons that was obtained by dividing the percentage of GABAergic neurons in the TagRFP-positive neurons by the percentage of total GABAergic neurons in the culture. A smaller value indicates a higher specificity towards glutamatergic neurons. The 2.1 kb of the mouse VGLUT1 promoter provided a significantly smaller ratio than all of the other promoters that were tested (*p* < 0.01, One-way ANOVA followed by Bonferroni’s test). The number of coverslips (n) analyzed in each group are represented in Table 1. (**d**) Quantification of TagRFP fluorescence intensity in neuronal soma. The synapsin I promoter, the VGLUT1 promoter and the Prox1 promoter provided a significantly higher expression in glutamatergic neurons than in GABAergic neurons (***p < 0.001, **p < 0.01, unpaired *t*-test).

Primary hippocampal cultures were prepared from *VGAT-Venus* Tg mice^15^, which express yellow fluorescent protein (Venus) specifically in inhibitory neurons, thus allowing discrimination of GABAergic neurons from glutamatergic neurons in hippocampal cultures^16^. To test the neuronal subtype-specificity of the promoters, red fluorescent protein (TagRFP) was expressed in the cultures using a pair of lentiviral vectors carrying the Tet-Off system under the control of each promoter (Fig. 1a). Although lentiviral vectors equipped with the human synapsin I promoter and the mouse α-CaMKII promoter showed selectivity toward glutamatergic neuron in the brain *in vivo*^5–7^, we found that these promoters caused reporter expression in a substantial fraction of GABAergic neurons when used in the cultured preparation (Fig. 1b).

We then attempted to identify a lentiviral promoter more specific to glutamatergic neurons in our culture system. We tested a 5′-upstream genomic region of three genes that are known to be expressed in hippocampal glutamatergic neurons. VGLUT1 is a common marker for presynaptic terminals of glutamatergic neurons in the hippocampus and the neocortex^17^. Dock10 (dedicator of cytokinesis 10) and Prox1 (prospero homeobox 1) have been used as markers for dentate gyrus granule cells (DGGCs), which send glutamatergic inputs to CA3 neurons^18,19^. Although DGGCs represent a limited fraction of hippocampal glutamatergic neurons, targeted gene expression in DGGCs in a culture preparation would be profitable for another line of studies because of their characteristic cellular functions, such as presynaptic plasticity in their mossy fiber boutons^20^. For an initial screening, roughly 2 kb regions of mouse genomic DNA including the TSS of these genes were used as putative promoter sequences (see the Materials and Methods section for details). All of the three sequences acted as promoters that provided efficient TagRFP expression in a subset of neurons. Notably, a 2.1 kb promoter for *VGLUT1* apparently restricted TagRFP expression to Venus-negative glutamatergic neurons (Fig. 1b).

The number of Venus-, TagRFP- and double-positive neurons were counted, and the percentage of total GABAergic neurons, of TagRFP-positive neurons and of GABAergic neurons in the TagRFP-positive population were estimated (Table 1). The synapsin I promoter and the α-CaMKII promoter drove TagRFP expression in more than 80% of the total neurons including a considerable fraction of GABAergic neurons (15.9 ± 1.6% and 23.3 ± 1.3%, respectively). The fraction of GABAergic neurons in TagRFP-positive cells following the use of the promoters was close to the fraction of GABAergic neurons that are intrinsically present in our cultures. In contrast, neurons expressing TagRFP upon the VGLUT1 promoter transduction contained only a small fraction of GABAergic neurons (4.2 ± 0.9%). However, the total fraction of TagRFP-positive neurons was also lower in these conditions. It should be noted that the fraction of GABAergic neurons that is intrinsic to our cultures substantially differed between the cultures and even between the experimental groups, making comparisons among the groups complicated. We, thus, introduced a ratio of TagRFP(+)-to-total GABAergic fraction by dividing the percentage of GABAergic neurons in the TagRFP-positive neurons (% of GABAergic in TagRFP(+)) by the percentage of total GABAergic neurons (% of GABAergic; Fig. 1c). A ratio that is closer to zero indicates a higher preference toward excitatory neurons, whereas a ratio that is closer to one indicates a lower preference toward excitatory neurons. The results indicate that the 2.1 kb VGLUT1 promoter conferred the highest preference for glutamatergic neurons over GABAergic neurons, while the 1.8 kb promoter of Dock10 and Prox1 worked neither as a DGGC-specific promoter nor as a glutamatergic-specific promoter (Fig. 1b,c). The TagRFP fluorescence intensity was also measured at the neuronal soma of both glutamatergic and GABAergic neurons (Fig. 1d). Of particular importance is that the VGLUT1 promoter showed the most significant difference in the expression level between the two neuronal types. Taken together, these results suggest that the promoter region of VGLUT1 is the best choice for a lentiviral promoter to restrict gene expression to glutamatergic neurons in primary hippocampal cultures.

**Table 1:** Quantification of reporter-expressing neurons and GABAergic neurons in neurons transduced by the five lentiviral promoters.

promoter	Number of coverslips analyzed	NeuN (+)	Venus (+)	TagRFP (+)	Venus & TagRFP (+)	% of GABAergic	% of TagRFP (+)	% of GABAergic in TagRFP (+)
Synapsin I	13	536 ± 10	117 ± 8	429 ± 12	69 ± 8	21.8 ± 1.5	80.0 ± 2.0	15.9 ± 1.6
α-CaMKII	7	539 ± 12	137 ± 6	475 ± 14	111 ± 8	25.5 ± 1.1	88.0 ± 1.3	23.3 ± 1.3
VGLUT1	12	542 ± 9	117 ± 7	202 ± 15	9 ± 2	21.7 ± 1.3	37.5 ± 3.0	4.2 ± 0.9
Dock10	4	549 ± 19	103 ± 9	202 ± 17	20 ± 5	18.7 ± 1.5	36.6 ± 2.1	9.7 ± 2.4
Prox1	6	518 ± 10	130 ± 5	364 ± 12	77 ± 4	25.0 ± 0.8	70.2 ± 1.5	21.3 ± 1.2

### A longer sequence of the VGLUT1 promoter reduced the transcriptional activity and a shorter sequence reduced the glutamatergic preference

**Figure 2:**
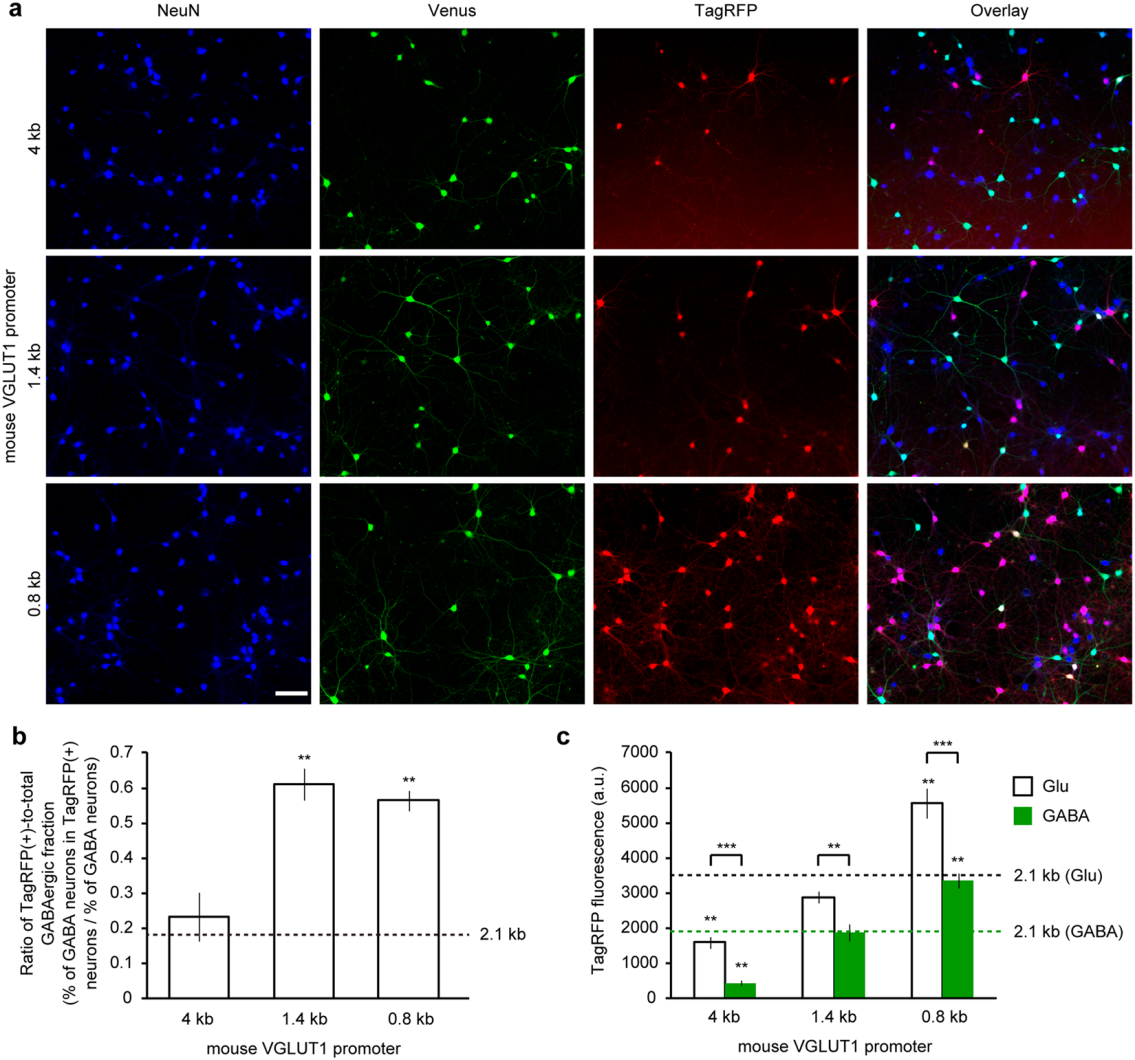
Glutamatergic neuron-specificity of varying lengths of the VGLUT1 promoter assessed in cultured hippocampal neurons from *VGAT-Venus* Tg mice. (**a**) Fluorescence images of cultured *VGAT-Venus* Tg neurons virally-expressing TagRFP by varying length of the VGLUT1 promoter. Neuronal somata are indicated by anti-NeuN immunostaining (blue). Venus fluorescence, amplified by anti-EGFP immunostaining, indicates GABAergic neurons (green). TagRFP fluorescence indicates reporter expression (red). Scale bar indicates 100 μm. (**b**) The ratio of TagRFP-positive populations within GABAergic neurons obtained from the varying lengths of the VGLUT1 promoter. The 1.4 kb and 0.8 kb segments of the VGLUT1 promoter provided a significantly larger value compared to the 2.1 kb segment, whose value is indicated by a dashed line. This suggests that the glutamatergic neuron-specificity was lower in the 1.4 kb and 0.8 kb segment condition (**p < 0.01, One-way ANOVA followed by Dunnett’s test). The number of coverslips (n) analyzed in each group are represented in Table 2. (**c**) Quantification of TagRFP fluorescence intensity in the neuronal soma. Both in glutamatergic and GABAergic neurons, the TagRFP expression obtained from the 4 kb segment of the VGLUT1 promoter was significantly decreased, while the TagRFP expression obtained from the 0.8 kb segment of the VGLUT1 promoter was significantly increased compared to that from the 2.1 kb segment of the VGLUT1 promoter (as indicated by the dashed line; **p < 0.01, One-way ANOVA followed by Dunnett’s test). In all of the conditions, the TagRFP expression was significantly lower in GABAergic neurons than in glutamatergic neurons (***p < 0.001, **p < 0.01, unpaired *t*-test).

The 2.1 kb VGLUT1 promoter in lentiviral vectors achieved highly preferential, but not exclusive, gene expression in cultured glutamatergic neurons. Next, we investigated whether a longer sequence of the promoter region would increase the specificity toward glutamatergic neurons. A 4 kb region of the 5′-upstream sequence of the *VGLUT1* gene, including the same downstream sequence from the TSS, was PCR amplified from the mouse genome and used as a promoter to express TagRFP in the cultured hippocampal neurons (Fig. 2a, Table 2). The ratio of the TagRFP(+)-to-total GABAergic fraction obtained by the elongated promoter was not improved compared to that obtained by the 2.1 kb promoter (Fig. 2b). Given that the fraction of TagRFP-positive neurons and the gene expression level were decreased by the use of a 4 kb sequence (Table 2, Fig. 2c), the 2.1 kb sequence is considered to be a better choice for the promoter.

**Table 2:** Quantification of reporter-expressing neurons and GABAergic neurons in neurons transduced by varying the lengths of the VGLUT1 promoter.

length of VGLUT1 promoter	Number of coverslips analyzed	NeuN (+)	Venus (+)	TagRFP (+)	Venus & TagRFP (+)	% of GABAergic	% of TagRFP (+)	% of GABAergic in TagRFP (+)
4 kb	5	567 ± 14	106 ± 5	109 ± 8	5 ± 1	18.8 ± 0.7	19.1 ± 0.9	4.5 ± 1.3
1.4 kb	8	523 ± 6	122 ± 9	168 ± 7	24 ± 3	23.4 ± 1.7	32.1 ± 1.0	14.6 ± 1.8
0.8 kb	8	515 ± 6	116 ± 6	288 ± 7	37 ± 3	22.4 ± 1.2	55.9 ± 1.4	12.7 ± 1.1

Decreasing the length of a promoter sequence leads to an enhancement of the potential applications. For instance, the use in AAV, which is another widely used viral vector that has a more limited capacity (4~5 kb). We investigated the effects of decreasing the promoter sequence length, examining how much of the 5′-upstream sequence in the VGLUT1 promoter is sufficient to restrict the expression of the TagRFP to glutamatergic neurons. By serially deleting the 5′-flanking region of the 2.1 kb promoter sequence, 1.4 kb and 0.8 kb fragments were isolated and used as a lentiviral promoter (Fig. 2a). As displayed in Fig. 2b, the shorter sequences resulted in an increased ratio of TagRFP(+)-to-total GABAergic fraction, indicating that at least 2.1 kb of 5′-upstream sequence of the *VGLUT1* gene was necessary to drive gene expression preferentially in glutamatergic neurons and to prevent expression in GABAergic neurons. Although the 0.8 kb fragment significantly increased the fraction of TagRFP-positive neurons, as well as the expression level of TagRFP in the soma (Table 2, Fig. 2c), we concluded that the 2.1 kb fragment of the VGLUT1 promoter was the most suitable sequence for use as a lentiviral promoter to target gene expression preferentially in glutamatergic neurons in primary hippocampal cultures.

### The efficacy of transgene expression by the VGLUT1 promoter increased during neuronal maturation

**Figure 3:**
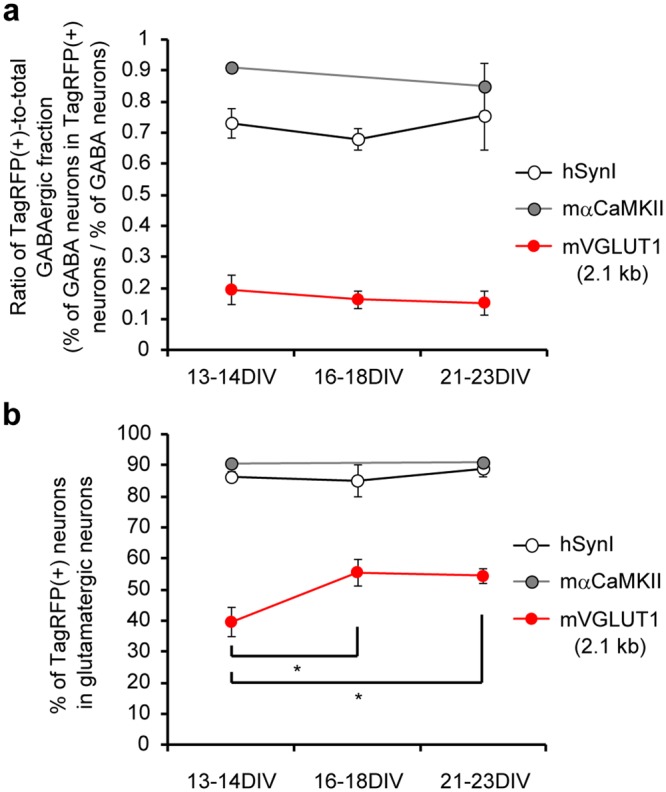
Developmental change in the cell type-specificity and reporter expression efficacy of the three different lentiviral promoters. (**a**) The ratio of TagRFP-positive populations within GABAergic neurons obtained using the synapsin I, α-CaMKII and 2.1 kb VGLUT1 promoters at three different stages in culture. There were no significant changes in any of the groups that were tested. (**b**) The percentage of TagRFP-expressing neurons in the glutamatergic (i.e., Venus-negative) population obtained using the synapsin I, α-CaMKII and 2.1 kb VGLUT1 promoters at three different stages in culture. The reporter expression efficacy of the VGLUT1 promoter significantly increased by the middle of the third week *in vitro* (*p < 0.05, One-way ANOVA followed by Bonferroni’s test).

The data described so far were obtained and pooled from cultured neurons at 13–18 days *in vitro* (DIV), at which time cultured neurons are still developing. We next tested whether neuronal maturation affects the pattern and efficacy of transgene expression, using neurons cultured over 3 weeks. The cultured neurons were transduced with lentiviral vectors at 7–12 DIV and subjected to the same experiments as the previously tested neurons. However, these cells were tested at 21–23 DIV not 13–18 DIV (Table 3). In addition, to further clarify the developmental changes, the pooled data that were obtained during 13–18 DIV were, wherever possible, divided into two categories; 13–14 DIV and 16–18 DIV. The ratios of the TagRFP(+)-to-total GABAergic fraction that were obtained using the synapsin I, α-CaMKII and 2.1 kb VGLUT1 promoters were plotted at three different culture stages (Fig. 3a). No significant changes were observed in any of the tested groups. This suggests that the cell type-specificity of these promoters did not critically depend on the developmental stages of the neuronal cultures within the range of stages that were under investigation. The glutamatergic (i.e., Venus-negative) neurons expressing TagRFP under the VGLUT1 promoter, however, significantly increased between 13–14 DIV and 16–18 DIV (Fig. 3b). This result likely corresponds to the upregulation in the expression level of VGLUT1 during postnatal weeks 2–3^17^. In contrast, the glutamatergic neurons expressing TagRFP under the synapsin I and α-CaMKII promoters remained unaltered, at ~90% in both groups (Fig. 3b). We also tested whether the transduction with the VGLUT1 promoter lentiviral vector at an earlier stage in culture improves the efficacy of reporter expression in glutamatergic neurons and the glutamatergic neuron-specificity. We found that both parameters were not significantly changed even if the cultures were transduced at 1 DIV (Supplementary Fig. 1).

**Table 3:** Quantification of reporter-expressing neurons and GABAergic neurons in neurons transduced by the three lentiviral promoters performed at 21–23 DIV.

promoter	Number of coverslips analyzed	NeuN (+)	Venus (+)	TagRFP (+)	Venus & TagRFP (+)	% of GABAergic	% of TagRFP (+)	% of GABAergic in TagRFP (+)
Synapsin I	5	567 ± 46	131 ± 12	473 ± 55	83 ± 16	23.1 ± 1.5	83.0 ± 4.8	17.0 ± 2.0
α-CaMKII	6	510 ± 39	114 ± 8	442 ± 27	84 ± 10	22.4 ± 0.6	87.4 ± 2.7	19.0 ± 1.8
2.1 kb VGLUT1	8	511 ± 15	120 ± 6	221 ± 13	9 ± 2	23.4 ± 0.9	43.3 ± 2.3	3.6 ± 0.8

### The VGLUT1 promoter did not drive gene expression in glial cells

**Figure 4:**
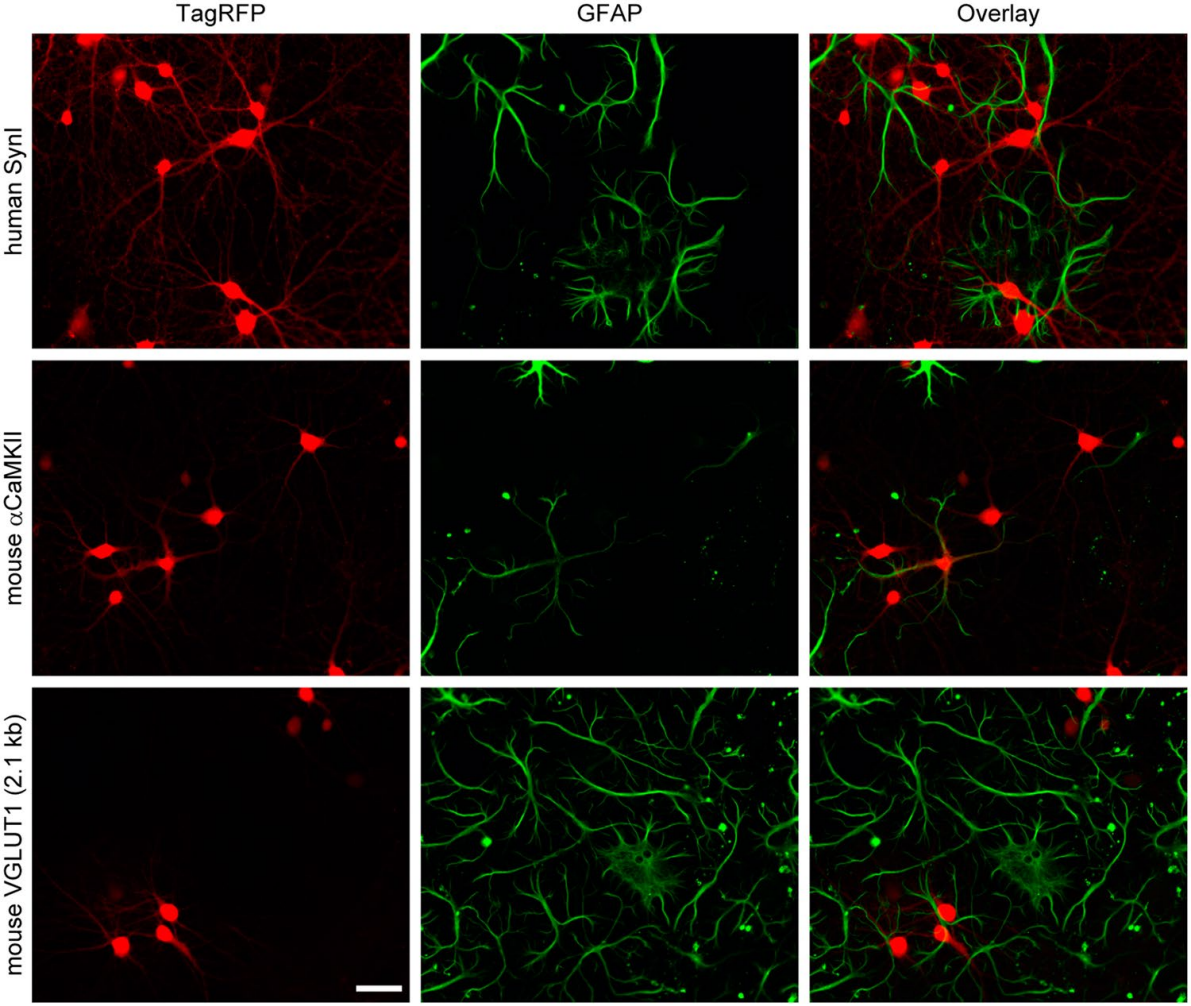
Neuronal cell-specificity of the three different lentiviral promoters assessed in a neuron-glia mixed culture. TagRFP fluorescence indicates reporter expression (red). Glial cells are labeled by anti-GFAP immunostaining (green). The α-CaMKII promoter produced TagRFP expression in a subset of glial cells, while the synapsin I promoter and the VGLUT1 promoter (2.1 kb) did not. Scale bar indicates 50 μm.

The preference for excitatory neurons of the VGLUT1 promoter was, thus far, investigated in primary neuron cultures that contained few glial cells. Therefore, it is not necessarily clear whether the gene expression is neuron-specific. To test this, we prepared neuron-glia mixed cultures by omitting the addition of a glial proliferation inhibitor and repeated the lentiviral transduction. Surprisingly, the α-CaMKII promoter caused TagRFP expression in a subset of glial cells that were identified by anti-GFAP immunolabeling (Fig. 4), although this promoter has long been used as neurons specific promoter *in vivo*. In contrast, as with the synapsin I promoter, the 2.1 kb VGLUT1 promoter did not produce any detectable TagRFP expression in glial cells (Fig. 4). These results indicate that the 2.1 kb VGLUT1 promoter is a neuron-specific promoter with a strong preference for excitatory neurons.

### The VGLUT1 promoter showed preference for the excitatory neurons expressing VGLUT1, but not VGLUT2

**Figure 5:**
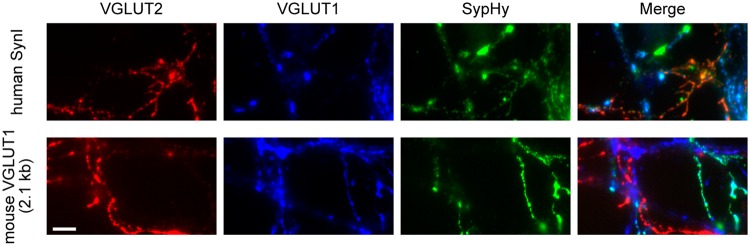
Specificity of the VGLUT1 promoter for excitatory neurons expressing VGLUT1, but not VGLUT2, assessed in mixed neuronal cultures of the hippocampus and brain stem. Fluorescence images of mixed cultures of the hippocampus and the brain stem prepared from wild type mice. Neurons express fluorescence-labeled synaptophysin (sypHy), whose fluorescence was amplified by anti-EGFP immunostaining (green). VGLUT1-positive and VGLUT2-positive synaptic boutons are visualized through immunostaining (blue and red, respectively). Although sypHy is co-localized both with VGLUT1-positive and VGLUT2-positive boutons in the synapsin I promoter condition (upper panel), sypHy is only co-localized with VGLUT1-positive boutons in the VGLUT1 promoter condition (lower panel).

Excitatory neurons in the CNS express either VGLUT1 or the other major isoform of VGLUT, VGLUT2. To test whether the VGLUT1 promoter showed specificity for VGLUT1-expressing neurons, we prepared mixed neuronal cultures of the hippocampus and brain stem, in which excitatory neurons predominantly express VGLUT1 and VGLUT2, respectively. To identify the transgene expression at the presynaptic terminals, neurons were transduced with lentiviral vectors that express sypHy, a fusion protein of rat synaptophysin and a pH-sensitive green fluorescent protein. Double-immunostaining with VGLUT1 and VGLUT2 antibodies indicated that the mixed cultures contained both VGLUT1-positive and VGLUT2-positive presynaptic boutons (Fig. 5). When the synapsin I promoter was used, sypHy was expressed at VGLUT1-positive, VGLUT2-positive and both-negative (possibly GABAergic) boutons (Fig. 5, upper panel). In contrast, the VGLUT1 promoter rarely induced sypHy expression at VGLUT2-positive boutons, while they were often co-localized with VGLUT1-positive boutons (Fig. 5, lower panel).

### The VGLUT1 promoter allows for preferential activation of excitatory neurons via optogenetic stimulation

**Figure 6:**
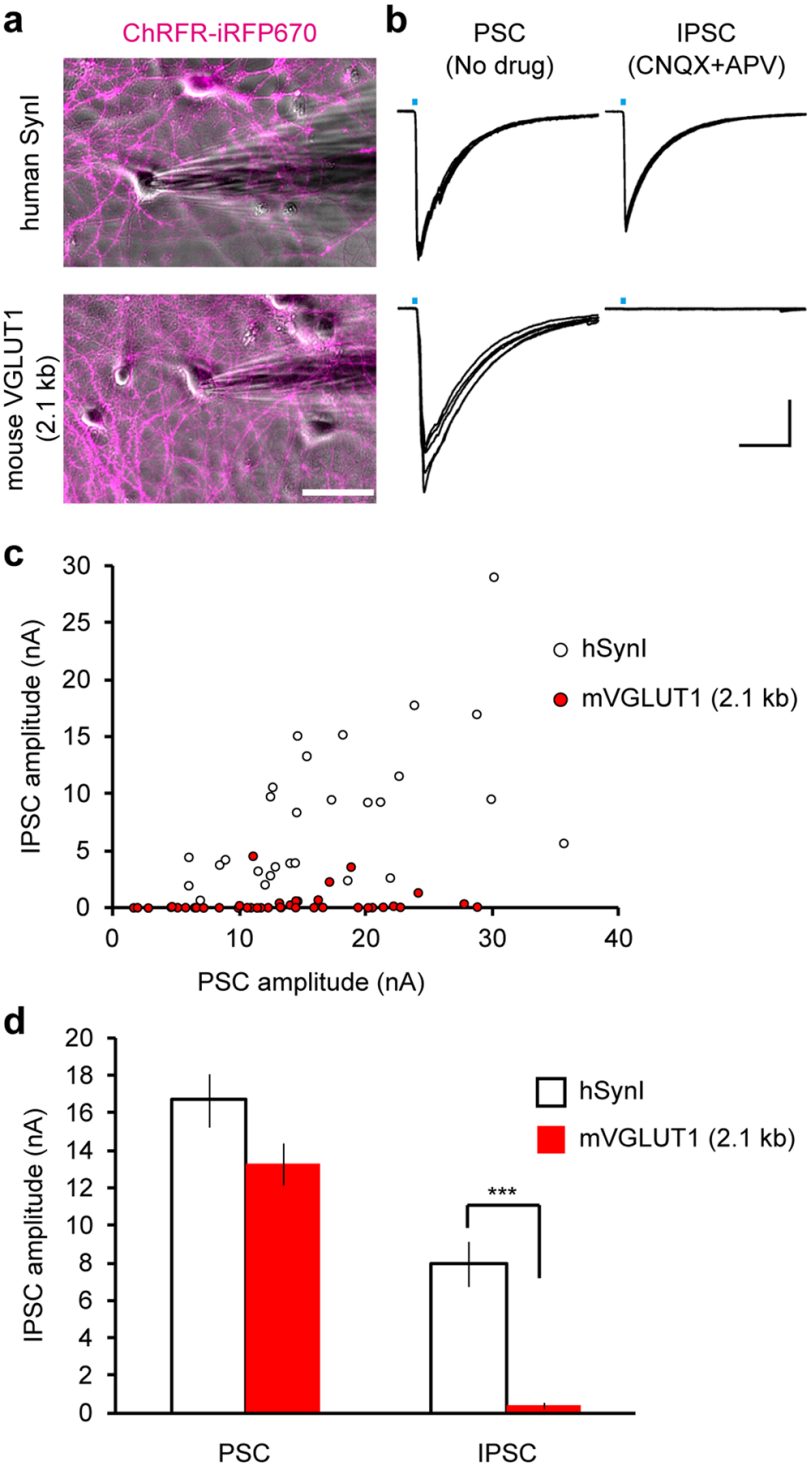
Optogenetic stimulation of cultured neurons transduced by the synapsin I promoter and the VGLUT1 promoter. (**a**) Fluorescence images of ChRFR-iRFP670 (magenta) overlaid on phase contrast images of the cultures transduced with lentiviral vectors equipped with the synapsin I promoter (upper panel) and the VGLUT1 promoter (lower panel). Patch clamp recordings were performed from ChRFR-negative neurons. Scale bar indicates 50 μm. (**b**) Representative traces of light-evoked currents recorded from the neuron shown in (**a**) in the absence of a postsynaptic receptor inhibitor (PSC, right) and in the presence of ionotropic glutamate receptor antagonists, CNQX and APV (IPSC, left). Five consecutive traces are overlaid. Light pulses of ~10 ms duration (blue bars) were applied through a 470 nm bandpass filter with an 11 nm bandwidth. Horizontal and vertical scale bars indicate 50 ms and 4 nA, respectively. (**c**) Summary plots displaying the amplitudes of the PSCs and the IPSCs recorded from the cultures that were transduced with lentiviral vectors equipped with the synapsin I promoter (white, n = 29 cells) and the VGLUT1 promoter (red, n = 40 cells). (**d**) The average amplitude of the PSCs and the IPSCs shown in (**c**). The IPSCs were almost entirely suppressed when the VGLUT1 promoter was used (***p < 0.001, unpaired *t*-test), while the PSCs recorded from the two groups were not significantly different (*p* = 0.06, unpaired *t*-test).

To further assess the neuronal subtype-specificity of the VGLUT1 promoter, we performed electrophysiological recordings in the transduced cultures. We exploited the light activation of channelrhodopsins to selectively stimulate transduced neurons. A chimeric channelrhodopsin, ChRFR, tagged with a fluorescent protein was expressed using the Tet-Off system driven by either the synapsin I promoter or the VGLUT1 promoter, and whole-cell voltage clamp recordings were performed from neurons devoid of ChRFR expression (Fig. 6a). In the absence of any postsynaptic receptor antagonist, a brief flash of blue light onto the cultures produced an inward postsynaptic current (PSC) in both groups (Fig. 6b). These PSCs can be composed of excitatory PSC (EPSC) and inhibitory PSC (IPSC) that are caused both monosynaptically and heterosynaptically. Because it is difficult to isolate monosynaptic EPSCs in neuronal mass cultures, due to the presence of concomitant heterosynaptic EPSCs that result in recurrent seizure-like activities, monosynaptic IPSCs were recorded in the presence of ionotropic glutamate receptor antagonists (CNQX and D-APV). In the cultures where ChRFR was expressed under the control of the synapsin I promoter, IPSCs were observed from almost all of the recorded neurons (Fig. 6b,c). This result was in agreement with the fact that the synapsin I promoter causes efficient gene expression in GABAergic neurons in the cultured hippocampus (Fig. 1). In contrast, in the cultures transduced with the VGLUT1 promoter lentiviral vector, no IPSCs were observed from the majority of the recorded neurons, indicating that the PSCs recorded in this culture mainly consist of monosynaptic EPSCs and subsequent heterosynaptic EPSCs/IPSCs. Only 4 of the 40 cells that were recorded from showed discernible, however relatively small, monosynaptic IPSCs (Fig. 6c). The average amplitude of the IPSCs was significantly smaller when the VGLUT1 promoter was used, whereas the average amplitude of the PSCs was not significantly different between the two groups (Fig. 6d). These results confirm that the VGLUT1 promoter (2.1 kb) combined with the Tet-Off system provided efficient gene expression with a strong preference for excitatory neurons in the culture preparations.

## Discussion

We characterized the neuronal subtype-specificity of five different lentiviral promoters, including two pre-existing sequences (synapsin I and α-CaMKII) and three newly-identified sequences (VGLUT1, Dock10, and Prox1), in cultured hippocampal neurons prepared from *VGAT-Venus* Tg mice. Despite their specificity towards excitatory neurons in the brain *in vivo*^5–7^, both the synapsin I and α-CaMKII promoters caused transgene expression in a large fraction (>70%) of GABAergic neurons in the culture preparations (Fig. 1c). The gene expression in GABAergic neurons following the use of a lentiviral vector equipped with the synapsin I promoter was, in fact, already evident from our previous study^16^, in which the luminal pH of synaptic vesicles could be monitored at both glutamatergic and GABAergic synapses present in the same culture. In this study, a significant difference in the vesicle pH dynamics between the two synapse types was uncovered. Because the monitoring of luminal pH of synaptic vesicles with pH-sensitive fluorescent proteins, such as pHluorin, has been widely used to visualize vesicle exo-endocytosis^21^, this result underscored the importance of the discrimination of glutamatergic neurons from GABAergic neurons. In the present study, we aimed to identify a lentiviral promoter that restricts transgene expression to glutamatergic neurons in cultures. Among the promoter sequences tested, 2.1 kb of the mouse VGLUT1 promoter region provided the most preferential gene expression in glutamatergic neurons, albeit with a small fraction (<20%) of weakly expressing GABAergic neurons (Fig. 1c). It should be noted that a population of DGGCs, which are primarily glutamatergic neurons, also express all of the markers of the GABAergic phenotype and co-release glutamate and GABA during development^22,23^. Because we counted the Venus-positive neurons from *VGAT-Venus* Tg mice as GABAergic neurons, these DGGCs might account for the fraction of GABAergic cells that expressed TagRFP by the 2.1 kb VGLUT1 promoter. In either case, the contribution of the TagRFP-positive GABAergic cells to the total of the TagRFP-positive neurons was trivial (~4%) in the cultured hippocampal neuronal population, where the GABAergic neuronal population was around 20% (Table 1). Moreover, the expression level observed in the GABAergic neurons was half as much as that observed in the glutamatergic neurons (Fig. 1d). In accordance with these results, light stimulation of the cultured neurons where chimeric channelrhodopsin was expressed by the VGLUT1 promoter rarely activated inhibitory neurons, whereas the cultures transduced with the synapsin I promoter lentiviral vector reliably showed light-evoked IPSCs (Fig. 6). Altogether, we conclude that the 2.1 kb VGLUT1 promoter can be used as a lentiviral promoter to effectively target gene expression to glutamatergic neurons in hippocampal cultures. We note that the Tet-Off inducible expression system is necessary to obtain adequate gene expression by the VGLUT1 promoter. When cultures were transduced with a lentiviral vector that expresses TagRFP directly from the 2.1 kb VGLUT1 promoter, no detectable TagRFP fluorescence was observed, at least, one week after the transduction (data not shown).

In hippocampal glutamatergic neurons, VGLUT1 plays an essential role in the filling of synaptic vesicles with glutamate and has been used as a reliable presynaptic marker of glutamatergic terminals^24^. The ectopic expression of VGLUT1 in non-glutamatergic neurons was sufficient to confer a glutamate-releasing phenotype^14^, thereby making its expression a hallmark of glutamatergic neurons. In fact, a previous study has reported that 11.6 kb of the mouse VGLUT1 promoter, consisting of 7 kb sequence upstream from the TSS and 4.6 kb of first intron, could be used as a viral promoter to drive gene expression mostly in VGLUT1-containing glutamatergic neurons when used in the *in vivo* brain^25,26^. The same group also reported that the preference for glutamatergic neurons was still retained using either the VGLUT1 upstream promoter (7 kb) or the first intron (4.6 kb), fused to a 323-bp basal promoter fragment that contained the TSS, although the reporter expression in inappropriate cell types (possibly GABAergic neurons) increased marginally^27^. In these studies, the relatively large promoter fragment was carried using Helper virus-free Herpes Simplex Virus Type 1 (HSV-1) plasmid vectors^28^. The use of these vectors, however, is still limited compared to other smaller viral vectors, due to the difficulty in obtaining large scale of high-titer stock of vector particles^29^. In the present study, we confined the VGLUT1 promoter region to a 2.1 kb sequence, which enabled the use of a lentiviral vector. Incidentally, the same authors have indicated that 2.4 kb of the phosphate-activated glutaminase (PAG) promoter in HSV-1 vectors could also act as a glutamatergic neuron-specific promoter when glutamatergic neurons were identified using PAG immunoreactivity^25^. While the specificity of the PAG promoter should be further evaluated, because PAG immunoreactivity is also observed in non-glutamatergic neurons^30,31^, the 2.4 kb PAG promoter may also have the potential to be used as a lentiviral promoter specific to glutamatergic neurons.

Our results are reasonably supported by recently advanced genome-wide databases of DNase-seq and Chip-seq (ChipAtlas; http://chip-atlas.org/). The analysis of the database of genomic sequences upstream of the *VGLUT1* gene (*Slc17A7*) revealed that both the binding sites of known transcription factors as well as the DNase-hypersensitive regions are highly clustered at a region from −2.0 kb to −1.0 kb of the *VGLUT1* TSS, indicating that this short segment is likely responsible for the transcriptional regulations of *VGLUT1*. Furthermore, both also appear in ~0.6 kb region surrounding the *VGLUT1* TSS. According to our results, it is therefore conceivable that the 0.8 kb fragment upstream of the *VGLUT1* contains the basal promoter that enables neuronal expression, whereas ~1.0 kb fragment between −2.0 kb and −1.0 kb might serve as an enhancer region that controls glutamatergic-selective gene expression. Additionally, the database shows that the binding site of a similar set of transcriptional factors are present in the first intron of *VGLUT1*, suggesting that the action of some of these transcription factors on the upstream segments, as well as the first intron, may cooperatively regulate the selective expression of VGLUT1. This is in line with a previous study that reported the importance of the first intron of *VGLUT1* in the selective gene expression in VGLUT1-expressing excitatory neurons^27^.

A disadvantage of the 2.1 kb VGLUT1 promoter found in our culture preparations was the low percentage of the reporter-expressing neurons (37.5–43.3% of total neurons; Tables 1 and 3), which roughly corresponds to the only half of the glutamatergic neurons in the cultures (Fig. 3b). Because the reporter-positive glutamatergic neurons increased developmentally (Fig. 3b), this result may be partly explained by the possibility that the developmental switch of VGLUT isoforms that is known to occur in the hippocampus, cortex, and cerebellum^32,33^ was still in progress during the early stages of our experiments (DIV 13–14). In mammals, there are three VGLUT isoforms: VGLUT1, VGLUT2 and VGLUT3^13,14,34–38^. While VGLUT3 is expressed in cells that are not traditionally considered to be glutamatergic, the expression of VGLUT1 and VGLUT2 covers the complete population of conventional glutamatergic neurons and the distribution of these isoforms is almost mutually exclusive in the adult brain^39^. However, it is known that, at early developmental stages VGLUT2 expression is more predominant in many brain regions, including the VGLUT1 expression domains. Additionally, VGLUT1 expression appears to be augmented only 2–3 weeks after birth^32,33^. Therefore, it is possible that the transcriptional activity of the VGLUT1 promoter is suppressed in a subset of the glutamatergic neurons at the early stages in culture. In support of this view, the VGLUT1 promoter did not drive gene expression in VGLUT2-containing glutamatergic neurons (Fig. 4), which are mainly located in the brain stem, thalamus, deep cerebellar nuclei and layer IV of the neocortex, with an additional sub-population of VGLUT2-containing neurons in the hippocampus^40^. Another possible reason for the lower expression efficacy by the 2.1 kb VGLUT1 promoter is the absence of the first intron, which may play an important role in transcriptional regulation^27^, as mentioned above.

In addition to the VGLUT1 promoter, we tested a 1.8 kb segment of the putative promoter sequence for the *Prox1* and *Dock10* gene, whose expression had been a marker of DGGCs^18,19^. Although DGGCs release glutamate, their cellular properties are largely different from pyramidal neurons in the CA1-CA3 region of the hippocampus. Thus, a DGGC specific promoter functional in cultured preparations would promote the comparative analysis of subpopulations in hippocampal glutamatergic neurons. However, our current attempt was not successful, as evident from the obvious expression observed in pyramidal neurons as well as GABAergic neurons following the use of both promoters (Fig. 1b). A further upstream sequence or the first intron may be required to obtain strict specificity to DGGCs, although the use of a longer fragment would restrict the use of such a promoter. Another subtype specific promoter that is desired to be established in cultured neurons is a GABAergic neurons-specific promoter. To the best of our knowledge, a 10.2 kb fragment of the glutamic acid decarboxylase (GAD) 67 (GAD67) promoter in a HSV-1 vector^25^, a 2.7 kb fragment of the GAD65 promoter in an AAV vector^41^ (but see^42,43^) and 2.6 kb fragments of the somatostatin and neuropeptide Y promoter from puffer fish in an AAV vector^43^ are known to be useful in targeting gene expression to GABAergic neurons in the adult rodent brain. Further investigation is required to confirm the cell type-specificity of these promoters in cultured preparations.

## Materials and Methods

### Plasmid construction

To express a transgene in cultured neurons using the Tet-Off inducible system, two lentiviral vectors were used^11^. A “regulator” vector expressed an advanced tetracycline transactivator (tTAad) under the control of the cell type-specific promoter, for which we tested genomic regions upstream of five different genes. A “response” vector expressed the transgene under the control of a modified tetracycline-response element (TRE) composite promoter. The “regulator” vector containing the human synapsin I promoter (0.4 kb) pLenti6PW-STB was generated as documented in a previous report^11^. The other “regulator” vectors were generated through the replacement of the synapsin I promoter in this vector with an alternative promoter sequences. To this end, pLenti6PW-STB was digested with EcoRI and the 78-bp fragment was removed to create pLenti6PW-STB2, in which the synapsin I promoter could be cleaved out through restriction digestion. The mouse α-CaMKII promoter (~1.3 kb) was amplified via PCR from FCK-Halo-GFP (a generous gift from Dr. M. Yuzaki, Tokyo Japan) using the following oligonucleotides: a forward primer (5′-AGACTCGAGTTAATTAACATTATGGCCTTAG-3′) and a reverse primer (5′-GGATCCCCCGCTGCCCCCAG-3′). The PCR product was digested using the restriction enzyme, XhoI, phosphorylated with T4 polynucleotide kinase (PNK) and cloned into the XhoI and EcoRV site of the pLenti6PW-STB2. Three different lengths (4,023 bp, 2,143 bp, and 1,443 bp) of putative promoter regions of the mouse *VGLUT1* gene, including 161 bp downstream of the TSS, were amplified via PCR from the mouse genomic DNA using the following oligonucleotides: the forward primers (5′-CTCCCCAGCTCCAGACAATG-3′) for the 4,023 bp fragment, (5′-TGTACAACCGCCAGACTTGT-3′) for the 2,143 bp fragment, (5′-CATTTTAGAGTTGGGGTTAC-3′) for the 1,443 bp fragment and a common reverse primer (5′-GACCCGCGTGGGCACAGCCACGAT-3′). The PCR products were phosphorylated with T4 PNK and cloned into the EcoRV site of the pLenti6PW-STB2 in an appropriate orientation. A 768 bp fragment of the putative VGLUT1 promoter was generated by digesting the 1,443 bp fragment with NheI. The product was then cloned into XbaI and the EcoRV site of the pLenti6PW-STB2. A 1,833 bp region of the mouse *Dock10* gene, including 107 bp downstream of TSS, was amplified via PCR from the mouse genomic DNA using the following oligonucleotides: a forward primer (5′-ATCCTTCCATACCAGCGGAT-3′) and a reverse primer (5′-ATCGCGGGCCGTGCCGCTC-3′). A 1,763-bp region of the mouse *Prox1* gene, including 296 bp downstream of TSS, was amplified via PCR from the mouse genomic DNA using the following oligonucleotides: a forward primer (5′-CACTGACACCGTTGTTCGAC-3′) and a reverse primer (5′-CGCACGCGGTGATGTCTTAC-3′). The PCR products of both the Dock10 and Prox1 putative promoter region were phosphorylated with T4 PNK and cloned into the EcoRV site of the pLenti6PW-STB2 in an appropriate orientation. We confirmed that the sequences of all of the PCR products were completely identical to the corresponding region in the reference database (GRCm38.p4 C57BL/6J). The “responsive” vectors were based on the pLenti6PW-TGB, which was also generated in a previous report^11^. DNA encoding TagRFP, sypHy and chimeric channelrhodopsin (ChRFR)^44^ that were tagged with either Venus or iRFP670 (ChRFR-Venus or ChRFR-iRFP670) were cloned into the vector by replacing the EGFP-encoding sequence.

### Lentiviral vector production

The lentiviral vectors were produced in HEK 293T cells as described previously^45^. HEK 293 T cells were transfected with 15 μg lentiviral backbone vector and helper plasmids (pCAG-kGP1 10 μg, pCAG4-RTR2 5 μg, and pCAG-VSVG 5 μg) using a calcium phosphate transfection method. Ten to 16 hrs after transfection, culture medium was replaced with fresh Neurobasal A medium (Gibco) supplemented with 2% B27 (Gibco) and 0.5 mM glutamine. Supernatants were collected 48 hrs after transfection and filtered to remove cell debris. Aliquots were flash frozen in liquid nitrogen and stored at −80 °C until use. The viral titer was estimated using the Lenti-X^TM^ qRT-PCR Titration Kit (Clontech) according to the manufacturer’s instructions. Primary neuronal cultures were transduced with titer-matched lentiviral vectors (1 × 10^7^ copies/dish) that were expected to be sufficient to transduce all of the neurons on the dish.

### Primary neuronal cultures

Primary hippocampal cultures were prepared from 0–1-day-old *VGAT-Venus* transgenic (Tg) mice^15^ or WT C57BL/6 mice, as described previously^45^. The hippocampus and brain stem mixed cultures were prepared from embryonic day 17–18 WT C57BL/6 mice. Briefly, harvested cells were plated onto poly-D-lysine-coated coverslips at a density of 25,000–30,000 cells/cm^2^ and kept in a 5% CO_2_ humidified incubator. At 3–4 days *in vitro* (DIV), 40 μM FUDR (Sigma) and 100 μM uridine (Sigma) were added to inhibit the growth of glial cells, unless otherwise noted. One-third of the culture medium was replaced with fresh medium every 2–4 days. Cultures were transduced with a pair of lentiviral vectors at 6–7 DIV and subjected to experiments at 13–18 DIV unless otherwise stated. Animals were treated according to our institutional guidelines for the care and use of animals (Doshisha University and Gunma University).

### Immunostaining

Neural cultures were fixed with 4% paraformaldehyde in phosphate buffer (Wako) for up to 1 hr at room temperature (RT) or overnight at 4 °C. After washing with PBS containing (in mM): 137 NaCl, 2.7 KCl, 10 Na_2_HPO_4_, and 1.76 KH_2_PO_4_ (pH ~7.3), permeabilization and blocking of non-specific binding were performed by incubating cells with PBS containing 5% fetal bovine serum (FBS) and 0.1% Triton X-100 for 20 min at RT. Cells were incubated with the following primary antibodies at a 1:1000 dilution in PBS containing 1% FBS for 3 hrs at RT: rabbit anti-NeuN (ab128886, Abcam), mouse anti-GFP (270F3, Synaptic Systems), rabbit anti-GFAP (ab7260, Abcam), rabbit anti-VGLUT1^36^ (Takamori *et al*., 2001) and guinea pig anti-VGLUT2^33^ (Miyazaki *et al*., 2004). Cells were rinsed three times with PBS for 5 min and incubated with the following secondary antibodies at a 1:1000 dilution in PBS for 1 hr at RT: Alexa 647- or Alexa 488-conjugated anti-rabbit IgG, Alexa 568-conjugated anti-guinea pig IgG or Alexa 488-conjugated anti-mouse IgG (Invitrogen). The fluorescence intensity of Alexa 488 was observed with 470/22 nm excitation and 514/30 nm emission filters. The fluorescence intensity of Alexa 647 was observed with 624/40 nm excitation and 692/40 nm emission filters. The fluorescence intensity of Alexa 568 and the TagRFP native fluorescence intensity in the immunostained preparation was observed through 556/20 nm excitation and 600/50 nm emission filters. The fluorescent images were acquired using inverted microscope (Olympus) equipped with a scientific cMOS camera (ANDOR) and 75-W xenon lamp.

### Electrophysiology

Primary hippocampal cultures prepared from WT mice, in which some neurons virally expressed ChRFR-Venus or ChRFR-iRFP670, were used for electrophysiological recordings at 13–15 DIV. Initially, ChRFR-Venus was used but this choice somewhat limited the experimental efficiency as fluorescent illumination of Venus was avoided before the electrophysiological recording to prevent ChRFR activation. The tagged fluorescent protein was then changed to iRFP670, as it possesses an excitation wavelength that is farther from the wavelength of the light required for ChRFR activation. The results obtained from these two fusion proteins were not significantly different and thus were pooled. Whole-cell voltage clamp recordings at a holding potential of −70 mV were performed in ChRFR-negative cells at room temperature (~25 °C) using a Multiclamp 700B amplifier (Molecular Devices) under the control of Clampex 10.2 (Molecular Devices). Neurons were continuously perfused with extracellular solution containing (in mM): 140 NaCl, 2.4 KCl, 10 HEPES, 4 CaCl_2_, 4 MgCl_2_, and 10 d-glucose (pH 7.3–7.4, 300–310 mOsm). The patch pipette internal solution contained (in mM): 136 KCl, 17.8 HEPES, 1 EGTA, 0.6 MgCl_2_, 4 ATP-Mg, 0.3 GTP-Na, 5 QX-314, 12 phosphocreatine, and 50 U/ml phosphocreatine kinase (pH 7.3–7.4, 290–300 mOsm). To activate ChRFR, a brief flash of light (~10 ms), passing through a 470/22-nm bandpass filter, was delivered through the objective. Light from a 75-W xenon lamp was gated with a Lambda 10–3 shutter (Sutter Instruments) under the control of Clampex 10.2. Inhibitory postsynaptic currents (IPCSs) were isolated by blocking excitatory transmission with 20 μM 6-cyano-7-nitroquinoxaline-2,3-dione (CNQX) and 25 μM D-2-Amino-5-Phosphonovaleric acid (D-APV). Data were digitized at 10 kHz and low-pass filtered at 3 kHz. Series resistance was compensated for by at least 70%. Recordings with series resistance <15 MΩ were analyzed using Clampfit 10.2 (Molecular Devices).

### Image analysis

The acquired images were analyzed using Fiji/ImageJ software. Regions of interest (ROIs) were defined depending on binarized image of NeuN immunostaining, which delineates the neuronal soma. More than 500 ROIs were obtained from every coverslip. These ROIs were transferred to images of Venus immunostaining and of TagRFP native fluorescence taken in the same field of view and used to count the number of Venus-, TagRFP- and double-positive neurons. The TagRFP fluorescence intensity was measured within the same ROIs on the TagRFP-positive neurons after background fluorescence was subtracted using a rolling ball algorithm.

### Statistical analysis

All data are shown as the mean ± standard error of the mean (SEM). Unpaired *t*-tests were applied to compare means of two experimental groups. For comparison of three or more groups, a one-way ANOVA followed by Bonferroni’s test was applied. For comparison of several groups against a control group, a one-way ANOVA followed by Dunnett’s test was applied. All statistical tests were two-tailed, and the level of statistical significance was indicated by asterisks: *p < 0.05, **p < 0.01, ***p < 0.001.

### Study approval

All experiments in this study were carried out in accordance of the regulations and guidelines of Doshisha University. Mice were housed in the animal facility in Doshisha University and were used under the approval of Doshisha University Animal Committee.

## Electronic supplementary material

Supplementary Information

## Data Availability

The data that support the findings of this study are available from the corresponding authors upon reasonable request.
