# Supplementary material for: Development of lentiviral vectors for efficient glutamatergic-selective gene expression in cultured hippocampal neurons
Source: Sci Rep. 2018 Oct 11;8:15156. doi: 10.1038/s41598-018-33509-5 (PMC6181963; doi:10.1038/s41598-018-33509-5)
Supplement: Supplementary file 1 — Supplementary Information [file 41598_2018_33509_MOESM1_ESM.pdf]

Supporting Information for

Development of lentiviral vectors for efficient glutamatergic-selective gene expression in cultured hippocampal neurons

Yoshihiro Egashira, Yasunori Mori, Yuchio Yanagawa & Shigeo Takamori

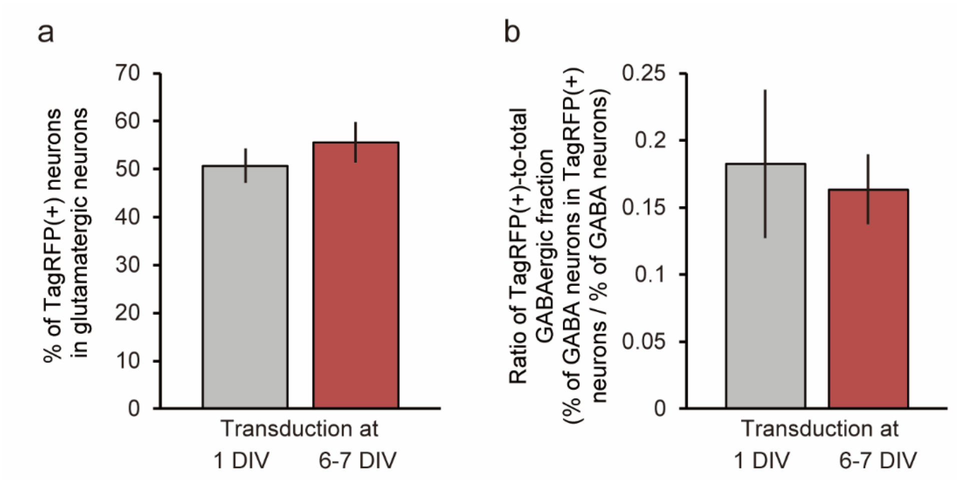

Supplementary Fig 1. Transduction with the VGLUT1 promoter (2.1 kb) lentiviral vector at an early stage in culture did not affect the efficacy of the reporter expression in glutamatergic neurons as well as the glutamatergic neuron-specificity. Cultures were transduced with lentiviral vectors at 1 DIV and TagRFP expression was analyzed at 17 DIV (n = 6 coverslips). Percent of the neuron expressing TagRFP in glutamatergic neurons (A) and the ratio of TagRFP-positive population within GABAergic neurons (B) were not different from those in the culture transduced at 6-7 DIV and fixed at 16-18 DIV (n = 5 coverslips) ($p = 0.40$ and 0.78, respectively, unpaired *t*-test).
